# Urinary Albumin-to-Creatinine Ratio (uACR) Point-of-Care (POC) Device with Seamless Data Transmission for Monitoring the Progression of Chronic Kidney Disease

**DOI:** 10.3390/bios15030145

**Published:** 2025-02-24

**Authors:** Artitaya Thiengsusuk, Napaporn Youngvises, Runtikan Pochairach, Rehab Osman Taha, Kridsada Sirisabhabhorn, Nadda Muhamad, Wanchai Meesiri, Wanna Chaijaroenkul, Kesara Na-Bangchang

**Affiliations:** 1 Drug Discovery and Development Center, Office of Advanced Science and Technology, Thammasat University, Pathum Thani 12120, Thailand; artitaya@tu.ac.th (A.T.); napaporn@tu.ac.th (N.Y.); runtikan@tu.ac.th (R.P.); 2 Graduate Program in Bioclinical Sciences, Chulabhorn International College of Medicine, Thammasat University, Pathum Thani 12120, Thailand; rehabos2@hotmail.com (R.O.T.); cwanna@tu.ac.th (W.C.); 3 Department of Medical Technology Laboratory, Thammasat University Hospital, Pathum Thani 12120, Thailand; kridsirimttu@gmail.com; 4 Department of Biomedicine and Health Informatics, Faculty of Pharmacy, Silpakorn University, Nakhon Pathom 73000, Thailand; muhamad_n@su.ac.th; 5 Bangkok High Lab Co., Ltd., Bang Khen District, Bangkok 10220, Thailand; wanchai.meesiri@bangkokhighlab.com; 6 Center of Excellence in Pharmacology and Molecular Biology of Malaria and Cholangiocarcinoma, Chulabhorn International College of Medicine, Thammasat University, Pathum Thani 12120, Thailand; 7 Chulabhorn International College of Medicine, Thammasat University, Pathum Thani 12120, Thailand

**Keywords:** albumin, albumin-to-creatinine ratio, chronic renal disease, creatinine, point-of-care medical device

## Abstract

Chronic kidney disease (CKD) continues to pose a critical global health challenge, making ongoing monitoring vital for effective management and preventing its progression to end-stage renal disease. The urinary albumin-to-creatinine ratio (uACR) stands out as a reliable biomarker. MyACR was developed and validated as a novel point-of-care (POC) device for identifying and monitoring the progress of CKD. MyACR device operates using a colorimetric-based spectroscopy to quantify albumin and creatinine levels at 625 nm and 515 nm, respectively. Calculated uACR values were compared with results from the reference turbidimetry method using a dataset of 103 random urine samples from patients at high risk of advanced CKD. The device showed excellent performance in detecting severe nephropathy, with sensitivity, specificity, and accuracy of 100%, 100%, and 100%, respectively. The PPV (positive predictive value) was 100%, indicating perfect identification of patients with severe nephropathy (uACR > 300 mg/g creatinine). The NPV (negative predictive value) was 100%, suggesting a strong ability to rule out severe nephropathy, though a small risk of false negatives remained. Bland–Altman analysis confirmed a high level of agreement, with 96.11% (for all data) and 95.87% (for uACR > 300 mg/g creatinine) of MyACR measurements falling within the 95% confidence interval (−27 to +19). Correlation analysis revealed a significant alignment between MyACR and the reference method (r^2^ 0.9720 to 0.9836). The ROC analysis suggested that combining uACR with the estimated glomerular filtration rate (eGFR) demonstrated strong predictive performance, yielding an area under the curve (AUC) of 0.933 (95% CI: 0.86–1.0). In conclusion, the MyACR device is a robust, affordable, and user-friendly tool for detecting nephropathy, showing performance comparable to the reference method. Its portability and cost-effectiveness make it particularly suitable for use in low-resource environments. Additionally, integrating uACR with eGFR enhances prognostic capabilities, offering a comprehensive approach to assessing kidney function and predicting CKD progression.

## 1. Introduction

Nephropathy has become an escalating global health challenge in recent years, with its prevalence and impact rising significantly [1,2]. Major contributing factors include diabetes, hypertension, and cardiovascular diseases, all of which can inflict severe kidney damage, ultimately leading to chronic kidney disease (CKD) [3]. CKD is characterized by persistent kidney damage or a glomerular filtration rate (GFR) below 60 mL/min per 1.73 m^2^ of body surface area for at least three months, regardless of the underlying cause. The disease’s progression is classified into stages based on GFR levels, with decreasing values indicating more advanced disease stages [1,4,5].

Macroalbuminuria, characterized by a urinary albumin-to-creatinine ratio (uACR) exceeding 300 mg/g creatinine (or albumin excretion over 300 mg/24 h), is a critical marker of CKD progression [6]. Its presence markedly elevates the risk of not only advancing kidney damage to end-stage CKD but also severe cardiovascular complications, including ischemic heart disease, stroke, peripheral vascular disease, and, in severe cases, even cancer [1,7]. Consequently, diligent monitoring of CKD progression is essential for effective disease management and timely intervention to mitigate these risks.

Currently, the most commonly used methods for the determination of uACR are immunoturbidimetry and enzyme-linked immunosorbent assay (ELISA) [8,9] for albumin and enzymatic assays [10,11] for creatinine. These methods are typically conducted using advanced automated machines in laboratory settings, which delay results and treatment access. While accurate, the methods are costly, require specialized and complex instrumentation, and necessitate the expertise of trained professionals, limiting their applicability in resource-constrained settings, such as community healthcare providers. To address these challenges, we have previously developed a point-of-care (POC) device capable of determining the uACR from a random urine sample. The device simultaneously measures urinary albumin and creatinine levels and calculates uACR, offering a more accessible and cost-effective option for CKD monitoring [12]. This POC device is designed to be accurate and simple to use, which enables rapid albuminuria detection and facilitates the ongoing tracking of CKD progression. In this study, we further assessed the clinical utility of this device for monitoring uACR in patients at high risk of progressing to kidney failure (CKD stage 5), particularly in resource-limited community healthcare settings and for home use. By offering a practical, affordable, and user-friendly alternative, this innovative test has the potential to revolutionize CKD management. It empowers healthcare professionals and patients to track disease progression more effectively, bridging critical gaps in care delivery even in underserved environments.

## 2. Materials and Methods

### 2.1. Chemicals and Reagents

Creatinine and tetrabromophenol blue (TBPB) were obtained from Sigma-Aldrich (St. Louis, MO, USA). Picric acid (PA) was sourced from Power Tech Chemical (Bangkok, Thailand). Human serum albumin (HSA) was acquired from Sisco Research Laboratories (Mumbai, India). Anhydrous sodium sulfate was supplied by QRëC (Auckland, New Zealand). Reagents such as trisodium citrate dihydrate, ammonium chloride, magnesium sulfate heptahydrate, and disodium oxalate were procured from Loba Chemie (Mumbai, India). Urea was purchased from Affymetrix (Santa Clara, CA, USA). Potassium chloride and sodium chloride were provided by Ajax Finechem (Wollongong, Australia). Sodium phosphate monobasic monohydrate was sourced from Carlo Erba (Emmendingen, Germany), while sodium phosphate dibasic heptahydrate and calcium chloride were obtained from Merck KGaA (Darmstadt, Germany). Sodium bicarbonate was acquired from VWR Life Science AMRESCO (Solon, OH, USA).

A stock solution of human serum albumin (HSA) and creatinine, at a concentration of 20 mg/mL, was prepared using deionized water and stored at 4 °C. Working solutions were prepared by diluting the stock solution with deionized water before use.

### 2.2. Instrumentation and Measurement Platform

MyACR is a portable, colorimetric-based spectroscopic point-of-care (POC) device designed to determine the urinary albumin-to-creatinine ratio (uACR) using an innovative dual optical sensor [12]. The test platform and operational schematic of the device are depicted in Supplementary Information Figure S1. To ensure adaptability across various environments, the MyACR device can function efficiently with multiple power options, including rechargeable lithium-ion batteries, standard AA/AAA alkaline batteries, or a standard AC power supply (110–240 V) via an adapter or plug system. This flexibility makes it ideal for diverse settings, from clinics with limited resources to home-based monitoring. The device simultaneously measures albumin and creatinine levels in urine and calculates the uACR value, displaying the results on its integrated screen. For enhanced utility, it is equipped with wireless connectivity, such as Bluetooth, enabling seamless data transmission to smartphones or computers. This feature supports healthcare providers with efficient data management and facilitates the creation of databases for advanced analysis and ongoing patient monitoring. To streamline operation, MyACR utilizes a built-in calibration curve to calculate uACR directly, eliminating manual calibration. Periodic calibration verification using a quality control (QC) sample ensures sustained test accuracy, making MyACR a reliable and user-friendly tool for CKD management.

MyACR uses two cuvettes (four transparent sides) with the outside dimensions of 7.5 × 12.5 × 45 mm (width × length × height) and a path length of 10 mm. It includes LEDs and photodiodes that measure light at two visible wavelengths, 625 nm and 515 nm, corresponding to each reaction of albumin and creatinine, respectively. The path length is the distance that the light passes through the solution inside the cuvette. The light (from both LEDs) simultaneously passes through both cuvettes.

The reaction between albumin and tetrabromophenol blue (TBPB) in a buffer at pH 3.2 forms a blue product, which absorbs light at 625 nm [13]. The concentration of albumin is calculated using the formula:

Albumin = [(A − intercept)/slope] × Dilution factor

where A is the absorbance at 625 nm, and the slope and intercept are derived from the linear calibration.

The reaction between creatinine and picric acid in a strong base forms a red complex that absorbs light at 515 nm [14]. The concentration of creatinine is calculated using the formula:

Creatinine = [(A − intercept)/slope] × Dilution factor

where A is the absorbance at 515 nm, and the slope and intercept are from the linear calibration.

The urine sample is placed into two separate vials. The first vial contains the TBPB reagent in buffer for measuring albumin, and the second vial contains picric acid in a strong base for measuring creatinine.

The uACR is calculated using the following equation:

uACR = (Albumin × 100)/Creatinine

The results are interpreted as follows:

- Normal urinary albumin if uACR < 30 mg/g creatinine
- Microalbuminuria if uACR = 30–300 mg/g creatinine
- Macroalbuminuria if uACR > 300 mg/g creatinine [15,16]

### 2.3. Clinical Application of MyACR

#### 2.3.1. Patients

The applicability of the MyACR device was validated using human urine samples collected from patients with uACR > 300 mg/g creatinine, i.e., CKD, diabetes, hypertension, and cardiovascular diseases. These patients were at high risk of progression to kidney failure (CKD stage 5) [17]. The test results were compared with the reference method (immunoturbidimetric) used at Thammasat Chalerm Prakiet Hospital, Prathum Thani Province, Thailand. The study was approved by the Ethics Committee of the Faculty of Medicine, Thammasat University (Project number MTU-EC-OO-2-1-168/66, approval number 266/2566). A minimum of 103 study subjects for patients at risk of renal failure were recruited. The required sample size was estimated using the formula:

$$n=\frac{\left[{\left(Z_{\frac{\propto }{2}}\right)}^{2}\times \left(1-P\right)\right]}{d^{2}}$$

where *n* = sample size, *Z* = 95% confidence interval (1.96), and *d* (%error) = 5%. *P* is the average sensitivity and specificity (0.95) of the uACR POC devices reported in previous studies [12].

#### 2.3.2. Albumin and Creatinine Assays

For the albumin assay, a reaction mixture consisting of 2 mL of tetrabromophenol blue (TBPB) solution (5 × 10^−5^ M) and 1% Triton X-100 in 0.05 M acetate buffer (pH 3.2) was combined with 3 mL of diluted urine sample. The urine sample was diluted with distilled water at suitable dilution, for example, 37.5 µL of the urine sample in 2962.5 µL of distilled water (1:80 dilution) [18]. Certain components in urine, such as urea, glucose, and some heavy metals, have been reported to interfere with the reactions. Increasing urine dilution avoids non-specific interference from these compounds at both wavelengths [18]. For the creatinine assay, an alkaline picrate reaction was prepared by mixing 1 mL of picric acid and 1 mL of sodium hydroxide, which was then mixed with the diluted urine sample in distilled water (1:80 dilution) and incubated at room temperature for 30 min before analysis [19].

The optical density (OD) of each sample mixture was recorded to measure the albumin and creatinine concentrations. The absorbance for albumin reaction was measured at 625 nm, and for creatinine, it was measured at 515 nm, using sample holders No. 1 and No. 2, respectively. The color intensity for each sample was adjusted by calculating the change in intensity (Δ intensity), which was obtained by subtracting the blank’s intensity from the sample’s, followed by correcting for the dilution factor (1:80).

#### 2.3.3. Calibration Curves

The urine analyses for albumin and creatinine using the MyACR system were calibrated based on standard concentration ranges. For albumin measurement, calibration was performed within a concentration range of 0–60 mg/L, while for creatinine, the calibration range was 0–2 mg/dL. These calibration curves were established to ensure accurate quantification of albumin and creatinine levels in urine samples, enhancing the reliability of the assay results.

#### 2.3.4. Validation of MyACR Performance

The performance of the MyACR POC device was assessed by comparing the measurements of albumin, creatinine, and uACR with those measured using the reference laboratory method. The standard cut-off value for severe condition of nephropathy was set as uACR > 300 mg/g creatinine [6]. The performance parameters evaluated included sensitivity, specificity, positive and negative predictive values, accuracy, false-positive and -negative rates calculated as follows:

- Sensitivity (%) = TP/(TP + FN)
- Specificity (%) = TN/(TN + FP)
- Positive Predictive Value (%) = TP/(TP + FP)
- Negative Predictive Value (%) = TN/(TN + FN)
- Accuracy (%) = (TP + TN)/(TP + TN + FP + FN)
- False-positive rate (%) = Number of renal failure cases misdiagnosed by MyACR/Total number of negative cases by the reference method
- False-negative rate (%) = Number of non-renal failure cases misdiagnosed by MyACR/Total number of positive cases by the reference method

where: TP = True Positive, TN = True Negative, FP = False Positive, FN = False Negative

To evaluate the consistency between the MyACR POC device and the reference laboratory method, a Bland–Altman plot was used to estimate the 95% confidence interval (95%CI) and the mean relative error.

#### 2.3.5. uACR Combined with eGRF as a Prognostic Marker of Severe Nephropathy

The Receiver Operating Characteristics (ROC) analysis was performed to assess the performance of uACR combined with the estimated glomerular filtration rate (eGFR). The true positive rate (sensitivity) was plotted against the false-positive rate (1-specificity) across various uACR levels [20]. The area under the curve (AUC) is a measure of the test’s overall performance. An AUC of 0.5 indicates a test that is no better than random chance, while an AUC of 1.0 indicates a perfect test

### 2.4. Statistical Analysis

Statistical analysis was performed using SPSS 25.0 software (SPSS: An IBM Company, Armonk, NY, USA). Qualitative data are presented as number and percentage (*n*, %) values. Quantitative variables are presented as median (range) and mean (standard deviation) values. The deviation from the mean for albumin, creatinine, and uACR values, as measured by the POC device, was calculated based on the values obtained from the reference laboratory method. This deviation is expressed as %DMV (percentage deviation from the mean value). The relationship between the concentrations of two quantitative variables was assessed using Spearman’s correlation test for non-normally distributed data. The Receiver Operating Characteristic (ROC) curve was applied to determine the clinical predictability of uACR combined with eGFR as a prognostic marker of severe nephropathy to advanced-stage CKD. A *p*-value of less than 0.05 was considered statistically significant (α = 0.05).

## 3. Results

### 3.1. Clinical Application of MyACR

Clinical application of MyACR was evaluated by comparing with the reference laboratory method using a single random urine sample obtained from 103 patients with a high risk of renal failure (52 males and 51 females, aged 18–97 years). Table 1 summarizes the demographics, clinical diagnosis, and kidney function parameters of all patients. Most patients were diagnosed with diabetes (DM), either alone or in conjunction with other diseases (*n* = 78), followed by hypertension (HT: *n* = 57), chronic kidney diseases (CKD: *n* = 44), and cardiovascular diseases (CVS: *n* = 4), either alone or in conjunction with other diseases.

Ninety-six (93.20%) cases had uACR (measured by reference method) > 300 mg/g creatinine, while only 7 (6.79%) had a level < 300 mg/g creatinine. Urinary albumin and creatinine concentrations, including uACR in those with >300 mg/g creatinine, ranged 207–5709 g/dL, 12–276 mg/dL, and 301–7602 mg/g creatinine, respectively. Eighty-nine (94.68%) patients had eGFR < 90 mL/min/1.73 m^2^.

#### 3.1.1. Calibration Curves

**Figure 1:**
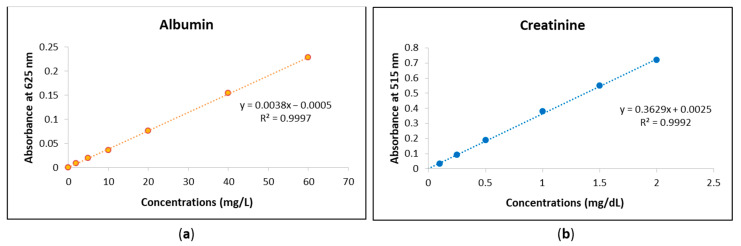
Calibration curves of (**a**) albumin at the concentration range 0−60 mg/L and (**b**) creatinine at the concentration range 0−2 mg/dL.

The urine analyses of albumin and creatinine using the MyACR system were calibrated to ensure accurate quantification of these biomarkers. The calibration curve for albumin was established within the concentration range of 0–60 mg/L, while the calibration range for creatinine was set between 0–2 mg/dL. To validate the accuracy and precision of the assay, standard solutions were prepared at multiple concentrations within these ranges. Each concentration point was measured in triplicate to confirm reproducibility and minimize variability. The calibration curves for both albumin and creatinine exhibited strong linear relationships, with correlation coefficients (r^2^) of 0.999 or higher (Figure 1a,b). This high degree of linearity indicates that the assay provides reliable and consistent measurements across the specified concentration ranges. The strong correlation suggests that the MyACR system effectively differentiates and quantifies albumin and creatinine levels in urine samples, ensuring the accuracy of subsequent sample analyses. Urine analyses of albumin and creatinine using MyACR were calibrated using the concentration ranges of 0–60 mg/L and 0–2 mg/dL, respectively. All calibration ranges yielded linear relationships with correlation coefficients (r^2^) of 0.999 or better (Figure 1a,b).

#### 3.1.2. MyACR Analysis Performance

To evaluate the analysis performance of MyACR in predicting the progress of nephropathy, the uACR values measured by MyACR were compared with those measured by the reference laboratory method using the cut-off value of >300 mg/g creatinine (Table 2). The sensitivity and specificity of MyACR were 100.00% (96/96) and 100% (7/7), respectively. The probability of correct renal failure diagnosis in a subject with uACR > 300 mg/g creatinine (positive predictive value: PPV) was 100% (96/96). The likelihood of a truly negative test result in a subject with uACR < 300 mg/g creatinine (negative predictive value: NPV) was 100.00% (7/7). The accuracy of MyACR expressed as a proportion of true positives and true negatives (correctly classified by MyACR) in all subjects was 100.00% (103/103). False positive and false negative rates were 0%.

#### 3.1.3. Test Agreement Analysis

**Figure 2:**
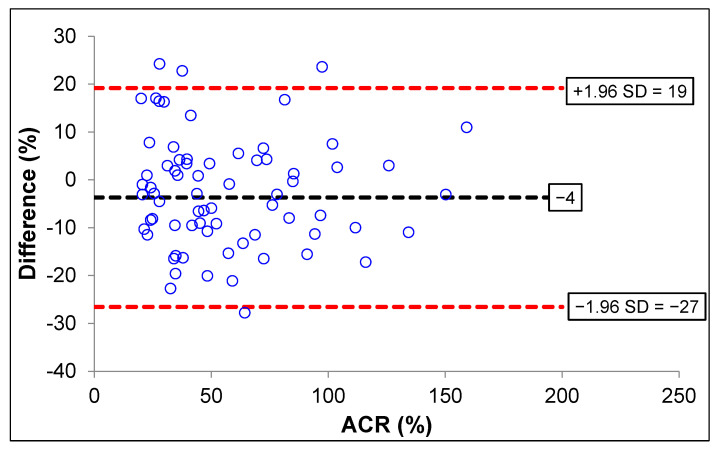
Bland–Altman plot analysis for accuracy of ACR measured by MyACR compared with the reference method.

The Bland–Altman plot analysis of the uACR was analyzed using MyACR compared with the reference method. Results showed that only four subjects with uACR > 300 mg/g creatinine had values outside the 95% CI (confidence interval) of the range of agreement limit (−27 to +19), suggesting that 95.87% of the values measured by the MyACR were in agreement with those measured by the reference method (Figure 2). When combining all data, four cases (96.11% agreement) had values outside the 95%CI.

#### 3.1.4. Correlation Analysis

**Figure 3:**
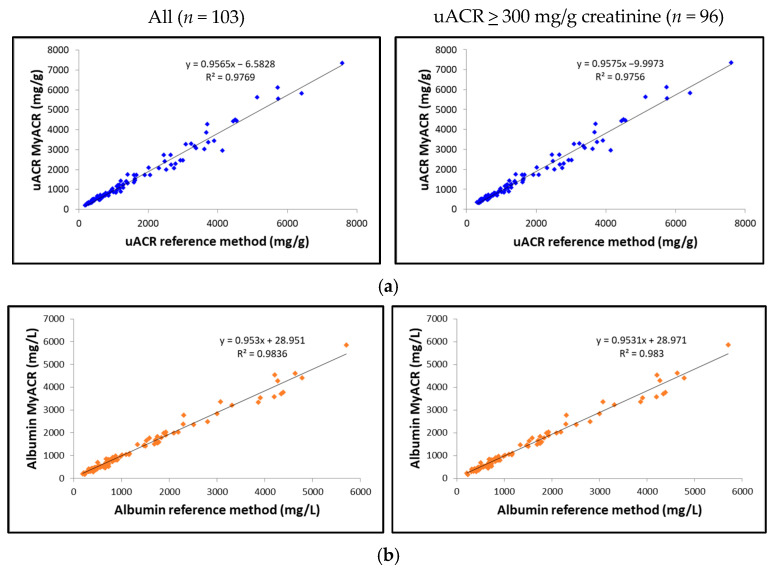

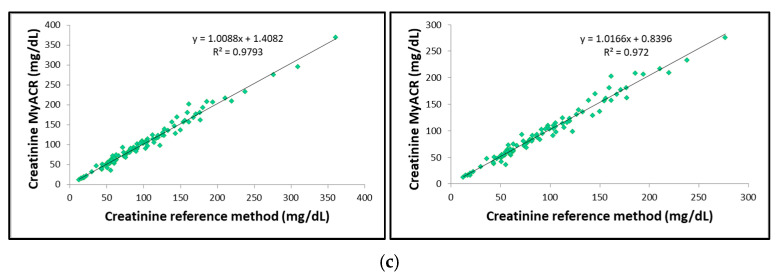
Correlation analysis of (**a**) uACR, (**b**) albumin, and (**c**) creatinine concentrations measured in random urine samples of patients with a high risk of chronic renal failure measured by MyACR compared with the reference method.

The correlation between the uACR, albumin, and creatinine values determined by MyACR and the reference method was analyzed. Results showed an excellent correlation between all parameters of the POC device MyACR and the reference method determined. The r^2^ in the samples with uACR > 300 mg/g creatinine ranged from 0.9720 to 0.9830, while those in all samples ranged from 0.9769 to 0.9836 (Figure 3a–c).

#### 3.1.5. uACR Combined with eGRF as a Prognostic Marker of Severe Nephropathy

**Figure 4:**
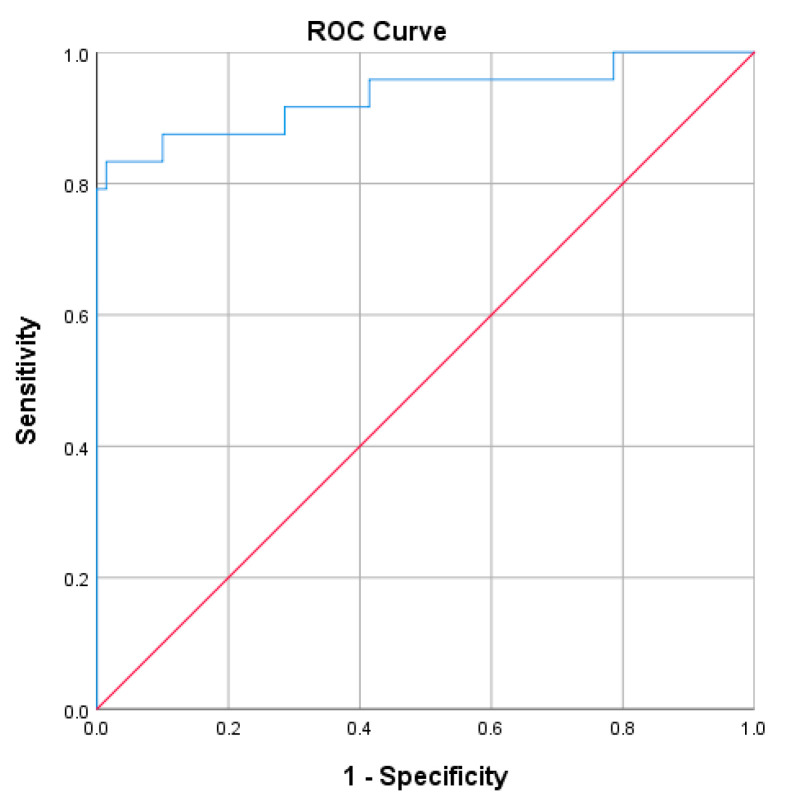
The ROC analysis of uACR (MyACR) combined with eGFR as a prognostic marker for the progression to advanced stage CKD.

The ROC analysis was performed to assess the performance of uACR combined with eGFR (uACR + eGFR) as a prognostic marker for the progression to advanced-stage CKD and renal failure (stage 5) (Figure 4). The AUC value was 0.933 (95%CI 0.86−1.0), which indicated that the combined test performed excellently in distinguishing between patients with severe nephropathy and those without. The small standard error (0.037) suggested that the AUC estimate was reliable. The *p*-value of 0.000 indicated that the AUC was statistically significant at α = 0.05.

## 4. Discussion

The MyACR device demonstrates exceptional performance in detecting severe nephropathy, achieving a sensitivity of 100% and a specificity of 100%. Its accuracy of 100% confirms the device’s reliability in distinguishing patients with severe nephropathy (uACR ≥ 300 mg/g creatinine) from those with normal or mildly impaired kidney function. A perfect PPV of 100% ensures all patients with significant kidney dysfunction are identified, while an NPV of 100% highlights strong but not absolute confidence in ruling out severe nephropathy, with a small risk of false negatives. The Bland–Altman analysis revealed that 96.11% (for all data), 95.87% (for uACR > 300 mg/g creatinine) of uACR values measured by MyACR were within the reference method’s 95% confidence interval (−27 to +19), underscoring the device’s consistency. Correlation analysis demonstrated outstanding accuracy, with r^2^ values exceeding 0.97 across all measurements. The device’s performance correlated strongly with clinical diagnoses of CKD and comorbid conditions, providing valuable insights into disease progression. For 74 CKD patients, 97.36% (*n* = 37) of those with CKD alone and 100% of those with CKD plus diabetes, hypertension, or other diseases (*n* = 17, 19, and 6, respectively) had uACR > 300 mg/g creatinine. Among 90 diabetes patients, 91.66% of those with diabetes alone (*n* = 44) and over 90% of those with additional conditions also showed severe nephropathy. Similarly, for 46 hypertension patients, 91.89% with hypertension alone and 100% with comorbidities had elevated uACR values. Patients with cardiovascular diseases alone or combined with other conditions consistently showed uACR > 300 mg/g creatinine. These findings highlight the MyACR device’s potential as a robust diagnostic tool for predicting nephropathy progression. Its accuracy, rapid results, and user-friendly design make it an invaluable resource for POC settings, especially in scenarios demanding swift and precise diagnostic capabilities. It was noted, however, that non-diseased controls (negative population) were not included in the study. Therefore, the spread of creatinine and albumin levels is unknown in the normal population. The estimates of sensitivity and selectivity are likely to be biased because of the small number of patients who had ACR < 300.

The estimated glomerular filtration rate (eGFR) is a crucial indicator of kidney function, calculated using variables such as serum creatinine levels, age, sex, and race. It evaluates how effectively the kidneys filter waste from the blood. A declining eGFR, particularly values below 60 mL/min/1.73 m^2^, is a hallmark of progressive kidney dysfunction and is widely used to stage chronic kidney disease (CKD) [5,21]. Studies have demonstrated that combining uACR and eGFR significantly enhances assessing severe nephropathy risk. Together, these biomarkers provide a more comprehensive prognostic tool than either one alone, aiding clinicians in identifying patients at heightened risk of end-stage renal disease (ESRD) or renal failure [22,23]. Our ROC analysis results corroborate the excellent diagnostic performance of uACR combined with eGFR in predicting severe nephropathy risk, underscoring its value as a powerful tool for early detection and proactive management of progressive kidney disease.

Commercially available POC devices for urinary albumin-to-creatinine ratio (uACR) include the Siemens CLINITEK Microalbumin Reagent Strips (sensitivity > 90% for early-stage CKD) [24], Nova Biomedical StatSensor Creatinine/ACR Meter [25], Abbott Afinion 2 Analyzer [26], and Quo-Test ACR Analyzer [27]. The Siemens CLINITEK utilizes urine test strips to detect microalbuminuria, with a measurable range for uACR from 20 to 2000 mg/g creatinine. Its sensitivity ranges from 80% to 90%, and specificity is 90–95%. However, its accuracy can be compromised by false positives and interference from non-albumin proteins, especially in patients with conditions such as diabetes and hypertension. The Nova Biomedical StatSensor directly measures creatinine and ACR using an electrochemical assay, with a uACR range of 10 to 1000 mg/g creatinine. It boasts sensitivity and specificity often exceeding 95%, but its performance can be influenced by variations in the urine sample matrix, requiring precise calibration for optimal accuracy. The Abbott Afinion 2 Analyzer, a portable device, uses immunoassay techniques to measure albumin and creatinine, calculating uACR over a range of 0.5 to 3000 mg/g creatinine. It offers a sensitivity and specificity of 90–95% and is favored in clinical settings for its rapid results and user-friendly operation. A key limitation is its relatively high cost, particularly for reagents, and the need for periodic maintenance. The Quo-Test ACR Analyzer measures albumin and creatinine to calculate uACR within a range of 2 to 2000 mg/g creatinine using an immunoturbidimetric assay, which is highly reliable for albumin detection. Its sensitivity and specificity range from 94% to 97% and 96%, respectively. However, like others, it is relatively expensive and requires calibration and maintenance for sustained accuracy.

The clinical applicability of the MyACR device is centered on monitoring uACR levels above 300 mg/g creatinine as a critical threshold for detecting the progression of severe nephropathy. MyACR effectively discriminates between patients with uACR > 300 mg/g creatinine and those below this threshold in individuals with chronic kidney disease (CKD). While the device is primarily designed for use with uACR values above this cut-off, its key advantages over other commercially available devices lie in its quantitative accuracy, cost-effectiveness, and suitability for resource-limited settings, particularly in developing countries. In addition to its affordability, MyACR enhances CKD management by incorporating real-time data transmission and patient tracking systems. These features facilitate better clinical decision-making and follow-up interventions, making the device a valuable tool in chronic kidney disease management. The device costs approximately USD 500, with each test costing around USD 2.5. In contrast, other POC devices on the market range in price from USD 1000 to 3000, with individual test costs varying from USD 1 to 25.

The performance of MyACR in detecting severe nephropathy positions it as a promising tool for routine screening and long-term monitoring of high-risk populations with CKD. Early detection of elevated uACR levels is crucial for identifying individuals at risk of progressive kidney disease. The ability to perform rapid testing in outpatient settings, such as primary care and community health clinics, would enable healthcare providers to detect early-stage kidney damage, facilitating prevention and timely intervention. While MyACR has shown excellent performance, further optimization could address the small proportion of false negatives observed in the analysis. Enhancing the device’s sensitivity for detecting low-grade nephropathy (microalbuminuria) and improving its ability to monitor declines in eGFR could significantly expand its clinical utility, providing even more comprehensive monitoring for CKD progression.

## 5. Conclusions

The MyACR device offers a highly reliable, cost-effective, and clinically valuable solution for detecting severe nephropathy. With excellent sensitivity, specificity, and accuracy, coupled with its affordability, MyACR stands out as a vital tool in addressing kidney disease, especially in resource-limited settings. When combined with eGFR, MyACR provides a robust diagnostic approach for predicting nephropathy progression, enabling clinicians to make timely, data-driven decisions to prevent further renal decline. Additionally, compared to existing POC devices, its performance underscores the utility of MyACR as an indispensable tool in modern nephrology.

## 6. Patents

The innovation patent ‘MyACR’ is approved by the Department of Intellectual Property of Thailand (No. 2302005386).

## Figures and Tables

**Table 1:** Demographics, urinary concentrations of albumin and creatinine, uACR values, eGFR, and clinical diagnoses of 103 patients with a high risk of renal failure.

Parameter		uACR (Reference Method)	
		<300	>300
Gender (*n* = 103)	Male (*n*, %)	7 (87.50%)	45 (47.40%)
	Female (*n*, %)	1 (12.50%)	50 (52.60%)
Age (year) (*n* = 103)	Mean (SD)	68.88 (9.96)	68.21 (14.61)
	Median (range)	69.00 (52.00–83.00)	68.00 (18.00–97.00)
Body weight (kg) (*n* = 103)	Mean (SD)	67.50 (11.59)	67.63 (16.86)
	Median (range)	69.50 (47.00–79.00)	65.00(34.50–118.00)
Height (cm) (*n* = 103)	Mean (SD)	166 (8.02)	161.89 (9.41)
	Median (range)	166.50 (155.00–178.00)	163.50 (140.00–182.00)
**Indication for uACR testing**			
**CKD (*n* = 44):**			
CKD only (*n* = 38)	(*n*, %)	1 (2.60%)	37 (97.40%)
CKD + CVD (*n* = 0)	(*n*, %)	0 (0.00%)	0 (0.00%)
CKD + others (*n* = 6)	(*n*, %)	0 (0.00%)	6 (100.00%)
**DM (*n* = 107):**			
DM only (*n* = 48)	(*n*, %)	4 (8.30%)	44 (91.70%)
DM + HT (*n* = 31)	(*n*, %)	3 (9.70%)	28 (90.30%)
DM + CKD (*n* = 17)	(*n*, %)	0 (0.00%)	17 (100.00%)
DM + CVD (*n* = 1)	(*n*, %)	0 (0.00%)	1 (100.00%)
DM + Others (*n* = 10)	(*n*, %)	0 (0.00%)	10 (100.00%)
**HT (*n* = 65):**			
HT only (*n* = 37)	(*n*, %)	3 (8.10%)	34 (91.90%)
HT + CKD (*n* = 19)	(*n*, %)	0 (0.00%)	19 (100.00%)
HT + CVD (*n* = 1)	(*n*, %)	0 (0.00%)	1 (100.00%)
HT + Others (*n* = 8)	(*n*, %)	0 (0.00%)	8 (100.00%)
**CVD (*n* = 4):**			
CVD only (*n* = 3)	(*n*, %)	0 (0.00%)	3 (100.00%)
CVD + others (*n* = 1)	(*n*, %)	0 (0.00%)	1 (100.00%)
**Kidney function parameters**			
Urinary albumin (g/dL) (*n* = 103)	Mean (SD)	449 (263)	1376 (1267)
	Median (range)	363 (188.00–921.00)	815 (207–5709)
Urinary creatinine (mg/dL) (*n* = 103)	Mean (SD)	188 (95)	94 (52)
	Median (range)	154 (91–361)	82 (12–276)
uACR (mg/g creatinine) (*n* = 103)	Mean (SD)	232 (32.05)	1744 (1567.27)
	Median (range)	220.50 (200–298)	1141 (301–7602)
Serum albumin (mg/dL) (*n* = 68)	Mean (SD)	4.40 (0.17)	3.86 (0.40)
	Median (range)	4.39 (4.19–4.69)	3.89 (2.68–4.54)
Creatinine in serum (mg/dL) (*n* = 103)	Mean (SD)	1.53 (0.69)	1.84 (1.04)
	Median (range)	1.50 (0.55–2.91)	1.52 (0.66–6.10)
BUN (mg/dL) (*n* = 84)	Mean (SD)	21.50 (5.60)	29.02 (14.92)
	Median (range)	20.00 (15.00–31.00)	24.50 (4.21–83.00)
eGFR:			
<90 mL/min/1.73 m2 (*n* = 89)	(*n*, %)	7(7.87%)	82 (92.13%)
≥90 mL/min/1.73 m^2^ (*n* = 5)	(*n*, %)	1 (20.00%)	4 (80.00%)

BUN = blood urea nitrogen, CKD = chronic kidney disease, CVD = cardiovascular diseases, DM = diabetes, HT = hypertension, eGFR = glomerular filtration rate.

**Table 2:** The assay performance of the POC device MyACR validated against the standard method based on a 300 mg/g creatinine cut-off value. Data are presented as the number of subjects (*n*) and percentage (%) values.

MyACR (*n*, %)	Reference Method (*n*, %)	
	ACR < 300	ACR ≥ 300
ACR < 300	7 (6.80%)	0 (0.00%)
ACR ≥ 300	0 (0.00%)	96 (93.2%)
Total	7	96

## Data Availability

Data are contained within the article.

## Supplementary Materials

The following supporting information can be downloaded at: https://www.mdpi.com/article/10.3390/bios15030145/s1. Figure S1. MyACR device and measurement platform.

## References

[1] Francis A., Harhay M.N., Ong A.C.M., Tummalapalli S.L., Ortiz A., Fogo A.B., Fliser D., Roy-Chaudhury P., Fontana M., Nangaku M. (2024). Chronic kidney disease and the global public health agenda: An international consensus. Nat. Rev. Nephrol..

[2] Neuen B.L., Chadban S.J., Demaio A.R., Johnson D.W., Perkovic V. (2017). Chronic kidney disease and the global NCDs agenda. BMJ Glob. Health.

[3] Monhart V. (2013). Hypertension and chronic kidney diseases. Cor Vasa.

[4] Feher M., Shaw K.M., Cummings M.H. (2005). Diabetes: Chronic Complications.

[5] Vaidya S.R., Aeddula N.R. Chronic Kidney Disease. https://www.ncbi.nlm.nih.gov/books/NBK535404/.

[6] Ansar M.M., ShahrokhiRad R., Lebady M.K. (2017). Risk Factors of Microalbuminuria and Macroalbuminuria in Type 2 Diabetic Patients in North of Iran—Rasht. Nephro-Urol. Mon..

[7] Jha V., Garcia-Garcia G., Iseki K., Li Z., Naicker S., Plattner B., Saran R., Wang A.Y., Yang C.W. (2013). Chronic kidney disease: Global dimension and perspectives. Lancet.

[8] Jensen M.B., Viken I., Høgh F., Jacobsen K.K. (2022). Quantification of urinary albumin and -creatinine: A comparison study of two analytical methods and their impact on albumin to creatinine ratio. Clin. Biochem..

[9] Martin H. (2011). Laboratory measurement of urine albumin and urine total protein in screening for proteinuria in chronic kidney disease. Clin. Biochem. Rev..

[10] Küme T., Sağlam B., Ergon C., Sisman A.R. (2018). Evaluation and comparison of Abbott Jaffe and enzymatic creatinine methods: Could the old method meet the new requirements?. J. Clin. Lab. Anal..

[11] Narimani R., Esmaeili M., Rasta S.H., Khosroshahi H.T., Mobed A. (2021). Trend in creatinine determining methods: Conventional methods to molecular-based methods. Anal. Sci. Adv..

[12] Muhamad N., Youngvises N., Plengsuriyakarn T., Meesiri W., Chaijaroenkul W., Na-Bangchang K. (2024). MyACR: A Point-of-Care Medical Device for Determination of Albumin-Creatinine Ratio (uACR) in Random Urine Samples as a Marker of Nephropathy. Diagnostics.

[13] Hashimoto M., Teshima N., Sakai T., Kato S. (2005). Colorimetric and Visual Methods for Urinary Protein Determination with Tetrabromophenol Blue. Bunseki Kagaku.

[14] Syal K., Banerjee D., Srinivasan A. (2013). Creatinine estimation and interference. Indian J. Clin. Biochem. IJCB.

[15] Committee A.D.A.P.P. (2023). 11. Chronic Kidney Disease and Risk Management: Standards of Care in Diabetes—2024. Diabetes Care.

[16] Keane W.F., Eknoyan G. (1999). Proteinuria, albuminuria, risk, assessment, detection, elimination (PARADE): A position paper of the National Kidney Foundation. Am. J. Kidney Dis. Off. J. Natl. Kidney Found..

[17] Levey A.S., Stevens L.A., Schmid C.H., Zhang Y.L., Castro A.F., Feldman H.I., Kusek J.W., Eggers P., Van Lente F., Greene T. (2009). A new equation to estimate glomerular filtration rate. Ann. Intern. Med..

[18] Ratanawimarnwong N., Ponhong K., Teshima N., Nacapricha D., Grudpan K., Sakai T., Motomizu S. (2012). Simultaneous injection effective mixing flow analysis of urinary albumin using dye-binding reaction. Talanta.

[19] Sittiwong J., Unob F. (2015). Detection of urinary creatinine using gold nanoparticles after solid phase extraction. Spectrochim. Acta Part A Mol. Biomol. Spectrosc..

[20] Nahm F.S. (2022). Receiver operating characteristic curve: Overview and practical use for clinicians. Korean J. Anesthesiol..

[21] Chen T.K., Knicely D.H., Grams M.E. (2019). Chronic Kidney Disease Diagnosis and Management: A Review. Jama.

[22] Hallan S.I., Ritz E., Lydersen S., Romundstad S., Kvenild K., Orth S.R. (2009). Combining GFR and albuminuria to classify CKD improves prediction of ESRD. J. Am. Soc. Nephrol. JASN.

[23] Ninomiya T., Perkovic V., de Galan B.E., Zoungas S., Pillai A., Jardine M., Patel A., Cass A., Neal B., Poulter N. (2009). Albuminuria and kidney function independently predict cardiovascular and renal outcomes in diabetes. J. Am. Soc. Nephrol. JASN.

[24] Mangili R., Deferrari G., Di Mario U., Giampietro O., Navalesi R., Nosadini R., Rigamonti G., Crepaldi G. (1992). Prevalence of Hypertension and Microalbuminuria in Adult Type 1 (Insulin-Dependent) Diabetic Patients Without Renal Failure in Italy—Validation of Screening Techniques to Detect Microalbuminuria. Acta Diabetol..

[25] Kosack C.M., Kieviet W., Bayrak K., Milovic A., Page A.L. (2015). Evaluation of the Nova StatSensor^®^ Xpress(TM) Creatinine point-of-care handheld analyzer. PLoS ONE.

[26] Radley A., Beer L., Rushdi D., Close H., McBurney S., Mackenzie A., Gourlay A., Barnett A., Grant A., Greig N. (1992). Implementation of point-of-care HbA1C instruments into community pharmacies: Initial development of a pathway for robust community testing. Ann. Clin. Biochem..

[27] Kateyam L., Sae-ung N., Anutrakulchai S., Cha-on U., Daduang J., Boonlakron S., CKDNET Group (2020). Effectiveness of URiSCAN 2 ACR strip test for albuminuria detection in screening of kidney disease. Arch. Allied Health Sci..

