# Supplementary figures and images for: Urinary Albumin-to-Creatinine Ratio (uACR) Point-of-Care (POC) Device with Seamless Data Transmission for Monitoring the Progression of Chronic Kidney Disease
Source: Biosensors (Basel). 2025 Feb 24;15(3):145. doi: 10.3390/bios15030145 (PMC11940007; doi:10.3390/bios15030145)

## Supplementary Information

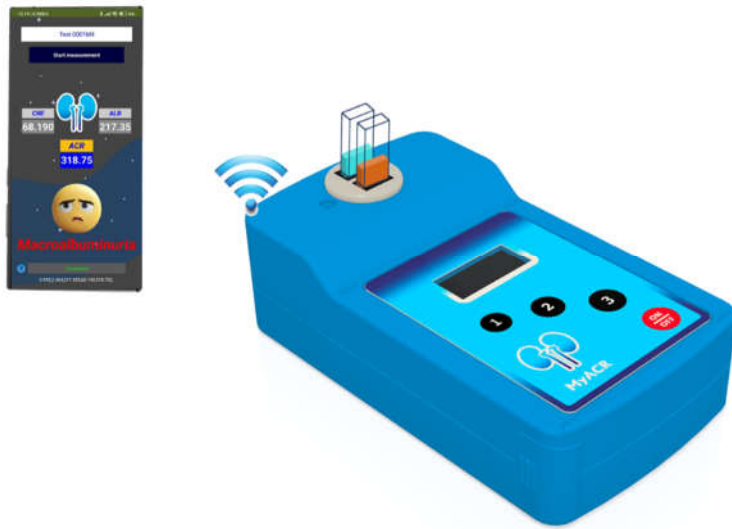

MyACR device and measurement platform.

Supplement: Supplementary file 1 [file biosensors-15-00145-s001.zip › biosensors-3472834-supplementary.pdf]
